# Does Microscope Assistance in Cold Steel Tonsillectomy Reduce the Risk of Postoperative Hemorrhage? Results of a Prospective Cohort Study

**DOI:** 10.1155/2017/8430907

**Published:** 2017-08-08

**Authors:** Thomas Wilhelm, Jan Wittlinger, Robert Georgiew, Christian Güldner, Stephan Hoch, Afshin Teymoortash, Thomas Günzel, Petar Stankovic

**Affiliations:** ^1^Department Otolaryngology, Head/Neck & Facial Plastic Surgery, Sana Kliniken Leipziger Land, Borna, Germany; ^2^Department Otolaryngology, Head and Neck Surgery, Philipps University of Marburg, Marburg, Germany; ^3^Department Otolaryngology, Head and Neck Surgery, Borromäus Hospital Leer, Leer, Germany

## Abstract

**Background:**

Posttonsillectomy hemorrhage (PTH) is the most feared complication. Dissection near the tonsillar capsule under microscopic view (TE_mic_) could be assumed to decrease PTH compared to traditional tonsillectomy (TE_trad_).

**Methods:**

In this study, patients were evaluated with respect to the need for surgical control (R/N: return/no return to theater (RTT): the day of surgery [0] or thereafter [1]). The findings at resection site and pain were measured.

**Results:**

869 patients were included (183 TE_mic_; 686 TE_trad_). PTH requiring RTT was not seen in the TE_mic_ group on the day of surgery (R0) while PTH requiring RTT subsequently (R1) was seen in 1.1% of the cases. In the TE_mic_ group, hemorrhages without a need for surgical control were observed in 0.6% (N0) and 3.4% (N1), respectively. The corresponding rates for TE_trad_ were as follows: R0, 0.3%; R1, 1.7%; N0, 0.6%; and N1, 3.6% (*p* > 0.05). Postoperative edema and local infection at resection site were proven to be predictive of PTH (*p* = 0.007).

**Conclusion:**

Microscope assistance in tonsillectomy did not statistically have an influence on the PTH even though there was a trend towards lower PTH rate in the TE_mic_ group. Benefit for TE_mic_ was observed in high-volume and long experienced surgeons.

## 1. Introduction

**Figure 1:**
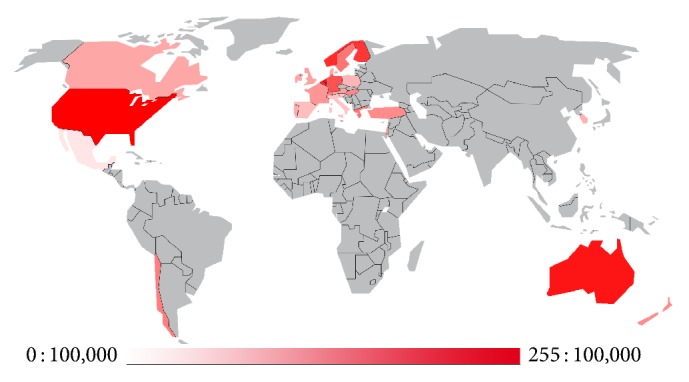
Tonsillectomies performed per 100,000 residents in the member countries of the OECD (mean: 128 : 100,000; median: 113 : 100,000; data originated between 2006 and 2014).

Tonsillectomy, adenoidectomy, paracentesis, and ventilation tube placement are the most common surgical procedures in otolaryngology. According to the data of the Organisation for Economic Co-operation and Development (OECD), tonsillectomy in developed countries is performed on average on 128 : 100,000 residents with a median of 113 : 100,000 and a range from 23 to 254 (Figure 1, http://stats.oecd.org/index.aspx?DataSetCode=HEALTH_STAT#  ⇨ Health Care Utilisation ⇨ Surgical procedures (shortlist) ⇨ Customize “Tonsillectomy”). This accounts for about 1.57 million procedures per year in the member countries (total population in the OECD in 2013: 1,257,114,000 inhabitants).

The most common consequences and complications of tonsillectomy are swallowing disorders, pain, and postoperative hemorrhage (PTH) which could be potentially life-threatening. According to the data of the Federal Office of Statistics in Germany, the rate of PTH is 5.98% in Germany, of which a sixth required return to the operating theater for revision surgery [1]. Therefore, in view of the annually performed procedures in the OECD, about 94,000 PTHs are to be expected. Fatal PTH is reported to range between 0.2 and 1.0 per 10,000 performed procedures resulting in an average of 0.9 : 10,000 [2–6].

Over the past four decades therefore, much effort has been made to minimize the risk of PTH in this high-volume surgical procedure. Various surgical techniques such as monopolar and bipolar dissection [7–10], CO_2_ laser [11], Coblation^©^ [12, 13], and BiClamp^©^ [14] have been developed and tested. The results of large-scale register studies, audits, and systematic reviews have shown that the hot techniques have proven to carry a higher risk of developing PTH (Table 1, [15–17]) and therefore the cold steel/cold hemostasis technique remains the “gold standard” [15]. On the other hand, a systematic review showed that PTH in children was not affected by the use of either the hot or the cold surgical technique [18].

In 1993, Andrea, from Lisbon, Portugal, recommended the use of a surgical microscope and bipolar diathermy dissection for tonsillectomy (TE) and stated the following advantages: intraoperative blood loss could be minimized, tissue trauma is limited, postoperative morbidity is lessened, operative time and associated support-service expenditures are reduced, and the use of the microscope provides unmatched lighting and visualization of the surgical field [19]. Andrea reported on 265 patients undergoing TE using this technique and experienced only 1 PTH. It is noteworthy to state that only 12% of the patients were adults and the majority were under 10 years of age. In the years that followed, other study groups picked up on the idea of microscope-assisted TE and reported their results, with PTH rates ranging from 0% to 7.1% [7, 20–23]. In these studies too, children constituted the majority (98.6%). In addition to the recommendations of the authors in these early studies, Windfuhr and colleagues more recently, in 2015, also recommended the use of a microscope to reduce PTH [24].

In 2008, the working group of Lee et al., from Busan, South Korea, published a study that evaluated vessel diameter 1 mm inside and 1 mm outside of the tonsillar capsule, as well as in the tonsillar capsule itself [25]: they found that the arterial and venous vessels increased significantly in diameter the further outside of the tonsils the vessels were located. Therefore, an anatomical basis could be established that supported the idea of coagulating and dividing the tonsil as closely as possible to the tonsillar capsule when performing a TE. Microscope assistance might be helpful in reaching this aim.

In our recent study, we aimed to clarify the benefits of microscope assistance in TE with regard to postoperative hemorrhage in a cohort of patients of various age groups (children and adults represented in a more balanced manner than in previous studies) and indications and compare the PTH rates to a group undergoing traditional cold steel TE.

## 2. Materials and Methods

In this retrospective analysis of two prospectively recorded cohorts (patients of all age groups), we compared the outcomes relating to postoperative hemorrhage and pain following cold steel TE with bipolar hemostasis without the use of optical tools (TE_trad_) to a second group where TE was performed with the assistance of a surgical microscope (TE_mic_).

The surgical technique did not differ from the traditional TE with cold steel (scissors and rasp). A surgical microscope (Zeiss OPMI Vario 700) with a varying magnification and autofocus was used. Bipolar coagulation (ERBE) for punctual hemostasis was used in both groups equally. Routine treatment included a pre- and intraoperative prophylaxis of vomiting and nausea with steroids. No antibiotics were administered routinely with the exception of cases with quinsy. Analgesics (paracetamol and ibuprofen) were weight-adjusted and administered daily for five to six days postoperatively.

The collection of patients' biographical data and laboratory coagulation screening were done routinely. Intraoperatively, the status of the tonsils (hyperplasia, scarring with the pharyngeal muscles) was judged by the surgeon. PTH and the need for return to theater (RTT) were noted, along with the inpatient postoperative day when those occurred. Pain expressed on a visual analog scale (0–100) was recorded in the morning and afternoon of each postoperative day while in hospital. The local wound conditions (edema, redness of the anterior pillars of the soft palate as a correlate with local inflammatory processes, and scab formation at the resection site) were recorded upon discharge from the hospital.

Posttonsillectomy hemorrhage (PTH) was classified in relation to the procedure date as 0/1 hemorrhage where 0 indicated PTH occurring on the day of the surgical procedure and 1 thereafter. Further breakdown focused on the need for surgical treatment and return to theater (R0/R1 versus N0/N1). All data in the primary data set was anonymized and subsequently analyzed by an external statistical institute (p-Wert, Jena, Germany, Mrs. H. Niggemann) according to national data protection laws.

Due to the exploratory character of the study, the statistical analysis focused on a description of the collected data. Categorical data were described by frequencies and continuous data were described by mean, standard deviation, median, minimum, and maximum. Using logistic regression, in which the surgeon was set as the random effect, two independent variables were compared with respect to the risk of PTH. Results are presented as odds ratios, 95% confidence intervals of odds ratios, and *p* values. This same method was used to evaluate the influence of microscope assistance on the risk of RTT in patients with bleeding. Results are presented separately for patients treated with microscope assistance and those treated without, as well as for the whole collective of patients. All statistical tests were two-sided. *p* values were calculated to enable the recognition of any statistically noteworthy findings and not in order to test a priori formulated null hypothesis. Stata/IC 13.1 for Windows was used for statistical analysis.

The study was approved by the Ethics Committee of the Saxonian Chamber of Physicians (EK-allg-9/16-1, 16.02.2016) and registered at the German Clinical Trials Register (http://www.drks.de, ID: DRKS00010076). All patients consented to the analysis of their anonymized data in a treatment contract.

## 3. Results

The data analyzed in this study were collected between 01.12.2003 and 15.12.2008 in a tertiary care center. Of 869 patients enrolled in the study, 686 patients were treated by conventional (“traditional”) cold steel tonsillectomy with punctual bipolar hemostasis (TE_trad_) and 183 patients by the cold steel technique/bipolar hemostasis with microscope assistance (TE_mic_). Demographic data are listed in Table 2: patients of all age groups were included. Indications for tonsillectomy are summarized in Table 2: 86.6% (TE_trad_) and 90.2% (TE_mic_) of the patients were referred for surgery due to hyperplasia or chronic or chronic-recurrent tonsillitis. At the time of the data collection, tonsillectomy was the treatment of choice in hyperplasia in Germany; this has changed in the recent years and now a partial tonsillectomy (tonsillotomy) would be performed.

In the preoperative laboratory testing, there was no difference in predicting postoperative hemorrhage with either the partial thromboplastin time (PTT; PTH yes versus no in TE_trad_: *p* = 0.759 and TE_mic_: *p* = 0.441) or the prothrombin time (PT; PTH yes versus no in TE_trad_: *p* = 0.247 and TE_mic_: *p* = 0.860).

**Figure 2:**
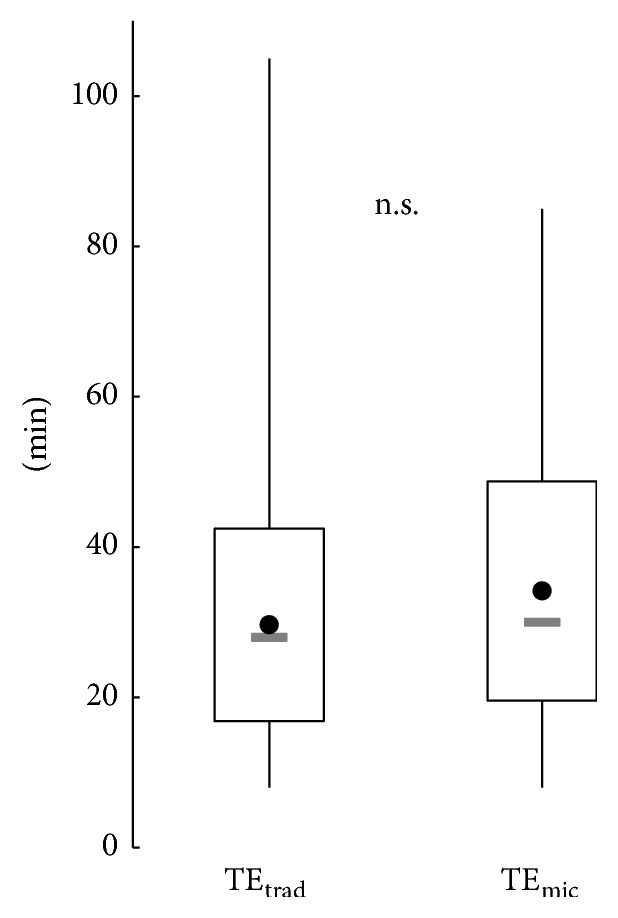
Procedural times in the study groups without the oncological cases (boxes represent mean ± standard deviation, whiskers represent the minimum/maximum, grey bars represent the median, and black circles represent the mean).

Procedural time did not differ between the groups (mean ± standard deviation, Figure 2): 29.7 ± 12.8 min (TE_trad_) versus 34.2 ± 14.6 min (TE_mic_). Intraoperatively, the surgeons judged the tonsils to be hyperplastic in 58.5% (*N* = 408/686) of the TE_trad_ group and in 63.4% (*N* = 116/183) of the TE_mic_ group. Scarring of the tonsils and the surrounding pharyngeal muscles was found in 73.9% (TE_trad_, *N* = 507/686) and 50.3% (TE_mic_, *N* = 92/183). An intraoperative quinsy was noticed in 12.8% of the TE_trad_ cases (*N* = 88/686) and in 4.9% of the TE_mic_ cases (*N* = 9/183).

The hospital stay did not differ between the groups: patients with traditional tonsillectomy stayed in hospital for 6.2 ± 1.3 days (mean ± standard deviation) and those treated with microscope assistance stayed for 6.4 ± 1.2 days.

**Figure 3:**
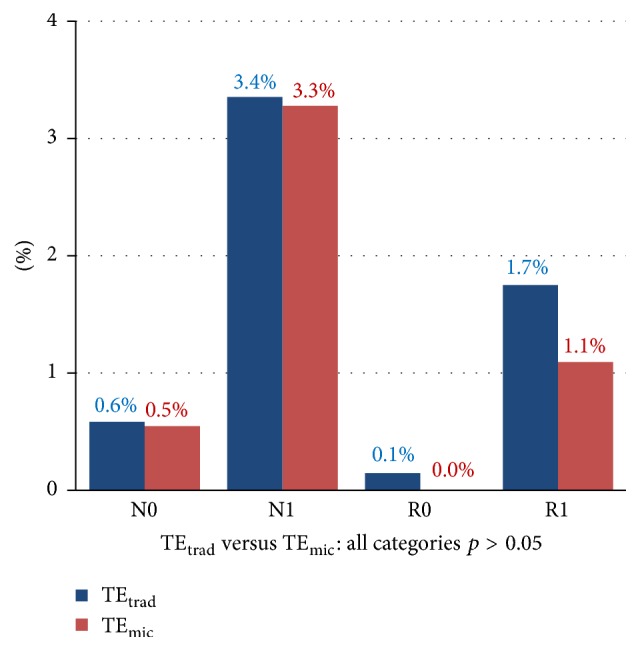
Rates of posttonsillectomy hemorrhage according to the time of bleeding and the need for surgical control (all *p* > 0.05).

In total, 49 posttonsillectomy hemorrhages (5.64%) were found in the groups combined: 40/686 (5.83%) were recorded in the TE_trad_ group and 9/183 (4.92%) were recorded in the TE_mic_ group (Table 3). When analyzed by the parameters of the time of bleeding (0/1) or the need for RTT (N/R), there was no significant statistical difference between the groups (*p* = 0.956). Even when the time of bleeding was not mentioned and the hemorrhage episodes were only analyzed on the basis of “RTT versus no RTT” (Table 3), there was no difference found by means of Fisher's exact test (*p* = 0.7491). The percentages of PTH for the different subsets (RTT/no RTT, 0/1) are displayed in Figure 3. An analysis by age group showed that the group most likely to develop postoperative hemorrhage through both techniques were adults from 19 to 65 years of age (Table 5).

In our study, we found a difference with respect to the professional experience of the surgeon (Table 4): the less experienced the surgeon (residents), the higher the PTH rate when using the microscope. In the TE_trad_ group, we found a PTH rate for the residents of 4.2% compared to 9.8% for consultants (odds ratio (OR): 0.40, 95% confidence interval (CI): 0.21–0.76, *p* = 0.0054); for the TE_mic_ group, the corresponding data are as follows: PTH residents 5.1% and consultants 4.8% (OR: 1.06, 95% CI: 0.28–4.10, *p* = 0.9283). When the data were analyzed by Fisher's exact test (Social Science Statistics: http://www.socscistatistics.com/Default.aspx), we found a difference in the PTH rates in TE_trad_ between residents (20 out of 480 patients, 4.17%) and consultants (20 out of 205 patients, 9.76%) with a *p* value of 0.0068; in the TE_mic_ group, there was no difference (residents 5 PTH (99): 5.1% versus consultants 4 PTH (84): 4.8%) as well as in the comparison of low- versus high-volume surgeons (volume set at 20; *p* > 0.05).

The PTH rate was only lowered when performing the microscope-assisted tonsillectomy in residents performing higher volumes of tonsillectomies. In the surgeons with long experience (consultants) and also those familiar with the use of the microscope, lower PTH rates were observed in microscope-assisted tonsillectomy.

**Figure 4:**
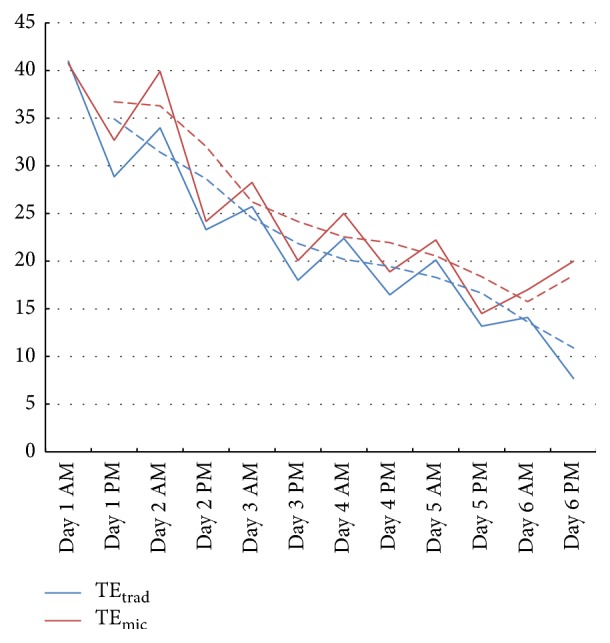
Postoperative mean pain scores (“pain during swallowing”) measured in the morning and afternoon by means of a visual analog scale (0–100). Dotted lines: trend line sliding average.

Postoperative pain, measured by means of an analog scale in the morning and afternoon for six consecutive days following surgery, showed no statistical difference (Figure 4).

When judging the local conditions at the resection site, we found postoperative redness of the anterior pillar of the soft palate as a correlate of local inflammatory processes at the resection site to be a predictive factor of PTH: OR for all patients was 3.07 (95% CI: 1.35–6.98), *p* = 0.007 and 2.97 (95% CI: 1.23–6.73), *p* = 0.015 for those operated on without the microscope. Postoperative edema was not predictive of PTH: OR = 1.46 (95% CI: 0.81–2.63), *p* = 0.203 and 1.57 (95% CI: 0.77–3.17), *p* = 0.214. Scab formation in the tonsillar beds was also not predictive of PTH.

## 4. Discussion

Hemorrhage following tonsillectomy, which is one of the most common procedures in otolaryngology, remains a challenge because it is potentially life-threatening and because of the need for surgical revision for bleeding control in 1% of cases [1]. Therefore, many efforts have been made to minimize the risk of posttonsillectomy hemorrhage (PTH). Primarily, different techniques have been adopted in order to prevent bleeding by proper hemostasis during primary surgery. Bipolar hemostasis and dissection, as well as monopolar dissection, were introduced decades ago [7, 16, 26]. More recently, lasers, Coblation, and harmonic scalpel have also been utilized for tonsillectomy [11, 12, 14, 27].

Large-scale audits and register studies from England and Sweden showed these “hot” techniques to increase the risk of postoperative hemorrhage [15–17]. Cold steel tonsillectomy with cold hemostasis (ties/packs and sutures, [24]) is therefore the “gold standard” in tonsillectomy in many countries. Nonetheless, tonsillectomy is still performed in most centers by the cold steel technique, while intraoperative hemostasis is ensured by means of bipolar coagulation. The latter has also been studied for decades and has proven safe and effective [26, 28]. Other authors have found the bipolar hemostasis or the power settings of such devices to be a risk factor for posttonsillectomy hemorrhage [24, 29].

As early as 1993, Andrea recommended the use of a surgical microscope for better visualization, more gentle dissection, and more precise hemostasis during tonsillectomy [19]. In his initial report, he reported only one PTH out of 265 patients operated on with this technique. It is crucial to note that the majority (87%) of subjects in that cohort were children and only 32 (12.5%) of the patients were adults. In the following years, other working groups reported their experience with microscope assistance in tonsillectomy in differently designed studies [7, 20–23], with average PTH rates of 2.02% (range: 0–7.14%). They also reported a significant decrease in blood loss, less pain, and a faster recovery from surgery. However, once again, as in the study of Andrea, 98.6% of the patients operated on were under 18 years of age.

In our study, we analyzed retrospectively data of two prospectively collected cohorts of all age groups and indications for tonsillectomy. In the majority of cases, chronic or chronic-recurrent tonsillitis was the indication for tonsillectomy. One cohort underwent tonsillectomy by the cold steel technique with bipolar coagulation for hemostasis (not suturing or ligatures in the tonsillar bed), whereas in the other cohort, a surgical microscope was additionally used for better visualization during cold steel tonsillectomy with bipolar hemostasis. We did not find a significant difference between the two groups relating to early and late PTH. We observed a slight trend towards lower PTH rates in the TE_mic_ group but the differences did not reach statistical significance. By subgroup analysis, we found high-volume surgeons and those surgeons with longer professional careers to benefit from microscope assistance. This might be explained by the fact that the young surgeon, in almost all cases, first cuts and then identifies the bleeder and thereafter coagulates whereas the experienced surgeon works on the principle of “identify-coagulate-cut.” Especially with microscope assistance, the early detection of small vessels has to be learned by young surgeons, whereas more experienced consultants directly benefit from the magnified visualization.

All in all, we found PTH rates in both groups and PTH requiring a return to theater comparable to other large-scale reviews regarding the same topic in Germany [1]. It could be assumed from this data that punctual bipolar hemostasis did not influence PTH rates markedly.

Despite the relatively large cohorts, the study proved to be underpowered, as in all other published studies to date. A power analysis based on our data (GPower, version 3.1.9.2.) with a *p* value set at 0.05 and an assumed power of 80% revealed that the number of patients that need to be treated is 7,046 in order to provide a statistically significant difference (Fisher's exact test, two-sided).

In 1993, Andrea also postulated less pain and faster recovery if microscope assistance was used for tonsillectomy [19]. Regarding postoperative pain, most recently, Schrötzlmair and colleagues found no difference between unaided eyes, loupes, and microscope assistance in tonsillectomy in a prospective randomized study using an intraindividual design in 45 patients [30]. These findings are hereby confirmed by the present study that also did not find a significant difference.

Interestingly, the local conditions at the resection site in our study proved to be significantly predictive of postoperative hemorrhage: local edema and redness of the anterior pillars of the soft palate as a correlate for local inflammatory processes were associated with a higher risk of posttonsillectomy hemorrhage.

## 5. Limitations of the Study

A major limitation of this study was the nonrandomized design responsible for the different sizes of the study groups. As a result of the open design, all age groups and indications were included, thus leading to very small groups in some indications. Nevertheless, chronic or chronic-recurrent tonsillitis was the leading indication for surgery in the majority of our study population.

Considering our results, the study was underpowered to detect a statistical difference between the PTH and RRT rates in the two study groups/tonsillectomy techniques.

## 6. Conclusion

Microscope assistance for the prevention of posttonsillectomy hemorrhage in the cold steel technique with punctual bipolar hemostasis could not reduce the PTH rate significantly. High-volume and experienced surgeons benefit the most from microscope assistance with regard to PTH since the microscope allows a magnified and illuminated surgical field, thus making it easier to identify vessels before cutting them. Local conditions at the resection site (edema, redness of the pillars) in the postoperative course could predict a higher risk of posttonsillectomy hemorrhage.

## Figures and Tables

**Table:** (a) RCS Tonsillectomy Audit, UK - 2005^(1)^

Surgery	*N*	Posttonsillectomy hemorrhage (PTH)						Return to operating theater for PTH					
		PTH		No PTH	OR	95% CI	*p* ^*∗*^	Return		No return	OR	95% CI	*p* ^*∗*^
		%	*N*	*N*				%	*N*	*N*			
Cold steel dissection & cold hemostasis	4.285	1.7	73	4.212	1			0.8	34	4.251	1		
Cold steel dissection & monopolar diathermy hemostasis	1.772	2.9	52	1.720	**1.7444**	**1.2169–2.5004**	**0,0025**	0.8	14	1.758	0.9957	0.5330–1.8600	0.9892
Cold steel dissection & bipolar diathermy hemostasis	11.956	2.7	323	11.633	**1.6021**	**1.2397–2.0704**	**0,0003**	0.7	84	11.872	0.8846	0.5930–1.3196	0.548
Monopolar diathermy forceps	452	6.6	30	422	**4.1018**	**2.6505–6.3478**	**<0.0001**	1.6	7	445	1.9668	0.8668–4.4626	0.1057
Bipolar diathermy forceps	10.240	4.6	473	9.767	**2.7943**	**2.1781–3.5847**	**<0.0001**	1.0	102	10.138	1.2579	0.8519–1.8576	0.2485
Bipolar diathermy scissors	2.322	5.1	119	2.203	**3.1167**	**2.3184–4.1900**	**<0.0001**	1.3	30	2.292	1.6365	0.9990–2.6809	0.0505
Coblation	1.565	4.6	72	1.493	**2.7825**	**1.9987–3.8737**	**<0.0001**	1.8	28	1.537	**2.2777**	**1.3766–3.7688**	**0.0014**
Other	1.329	4.1	55	1.274	**2.4909**	**1.7456–3.5544**	**<0.0001**	1.4	19	1.310	**1.8134**	**1.0309–3.1900**	**0.0389**

Total	33.921	3.5	1.197	32.724				0.9	318	33.603			

**Table:** (b) National Tonsil Surgery Register, Sweden, 2014^(2)^

Surgery	*N*	Late posttonsillectomy hemorrhage (PTH)						Return to operating theater for late PTH^*∗∗*^					
		PTH		No PTH	OR	95% CI	*p* ^*∗*^	Return		No return	OR	95% CI	*p* ^*∗*^
		%	*N*	*N*				%	*N*	*N*			
Cold steel dissection & cold hemostasis	636	3.3	21	615	1			1.1	7	629	1		
Cold steel dissection & hot hemostasis	6.406	8.8	566	5.840	**2.8383**	**1.8217–4.4222**	**<0.0001**	2.7	171	6.235	**2.4644**	**1.1522–5.2709**	**0.0201**
Bipolar diathermy scissors	1.314	13.4	176	1.138	**4.5292**	**2.8506–7.1963**	**<0.0001**	3.2	42	1.272	**2.967**	**1.3254–6.6419**	**0.0082**
Coblation	902	9.9	89	813	**3.2059**	**1.9701–5.2169**	**<0.0001**	2.4	22	880	2.2464	0.9538–5.2910	0.0641
Ultracision	259	16.2	42	217	**5.6682**	**3.2827–9.7873**	**<0.0001**	3.9	10	249	**3.6087**	**1.3585–9.5863**	**0.01**

Total	9.517	9.4	894	8.623				2.6	252	9.265			

^(1)^Breakdown according to surgery among all 33,921 patients who underwent tonsillectomy in the RCS Tonsillectomy Audit, UK, 2005. ^(2)^Breakdown according to surgery among the 9,603 responders to the 30-day questionnaire (out of 15,734 patients who underwent tonsillectomy) in the National Tonsil Surgery Register, Sweden, 2014. RCS: Royal College of Surgeons; OR: odds ratio; CI: confidence interval; ^*∗*^*z* statistic; odds ratios were calculated with MedCalc online: https://www.medcalc.org/calc/odds_ratio.php. ^*∗∗*^Given some inconsistencies in the Swedish paper, the following assumptions were made: (1) the figures for the number of patients who answered the 30-day questionnaire in each of the individual surgery-type groups were correct; (2) the figures for the “yes outcomes” (“yes PTH” and “yes RTT”) were correct; and (3) the figures for the “no outcomes” (“no PTH” and “no RTT”) consisted in the difference between the numbers of those who answered the 30-day questionnaire and those for the “yes answers.”

**Table 2:** Demographic data, age groups, and indications for tonsillectomy of the included patients are listed.

	TE_trad_	TE_mic_
*N*	686	183
M : F	269 : 417	82 : 101
Mean age ± SD		
Male	26.4 ± 16.6	23.9 ± 17.6
Female	25.1 ± 12.8	19.7 ± 13.9
Age groups: *N* (%)		
0–5	37 (5.4%)	29 (15.8%)
6–10	43 (6.3%)	27 (14.8%)
11–18	169 (24.6%)	34 (18.6%)
19–45	369 (53.8%)	79 (43.2%)
46–65	56 (8.2%)	10 (5.5%)
>65	12 (1.7%)	4 (2.2%)
Indication: *N* (%)		
Hyperplasia	24 (3.5%)	5 (2.7%)
Acute tonsillitis	3 (0.4%)	1 (0.5%)
Mononucleosis	9 (1.3%)	1 (0.5%)
Quinsy	71 (10.3%)	8 (4.4%)
Chronic tonsillitis	274 (39.9%)	51 (27.9%)
Chronic-recurrent tonsillitis	296 (43.1%)	109 (59.6%)
Cervical cyst	1 (0.1%)	0 (0.0%)
Obstructive sleep disorders	4 (0.6%)	4 (2.2%)
Oncological cases	4 (0.6%)	4 (2.2%)

**Table 3:** PTH rates stratified by postoperative time (0: on the day of surgery, 1: after the day of surgery) and the need for a surgical control (N: no return to theater, R: return to theater). *p* values are calculated by Fisher's exact test.

	TE_trad_ *N* = 686		TE_mic_ *N* = 183		Total *N* = 869		*p* value^*∗*^
No PTH	646	94.2%	174	95.1%	820	94.4%	0.7212
N0	4	0.6%	1	0.6%	5	0.6%	1.0000
N1	23	3.4%	6	3.3%	29	3.3%	1.0000
R0	2	0.3%	0	0.0%	2	0.2%	1.0000
R1	11	1.6%	2	1.1%	13	1.5%	1.0000

N0 + N1	27	3.9%	7	3.8%	34	3.9%	1.0000
R0 + R1	13	1.9%	2	1.1%	15	1.7%	0.7491

^*∗*^Fisher's exact test.

**Table 4:** Rates of posttonsillectomy hemorrhage (PTH), displayed by the professional experience of the surgeons.

Surgeon's PE^*∗*^	TE_trad_ *N* = 686		TE_mic_ *N* = 183		All cases *N* = 869	
*res*	*1 (1)*	*100%*	*0*	*0%*	*1 (1)*	*100%*
*res*	*0*	*0%*	*0 (9)*	*0%*	*0 (9)*	*0%*
*res*	*0*	*0%*	*0 (11)*	*0%*	*0 (11)*	*0%*
res	14 (263)	5.3%	2 (30)	6.7%	16 (293)	5.5%
res	2 (83)	2.4%	2 (31)	6.5%	4 (114)	3.5%
res	3 (134)	2.2%	1 (18)	5.6%	4 (152)	2.6%

*con*	*4 (26)*	*15.4%*	*0 (2)*	*0%*	*4 (28)*	*14.3%*
*con*	*0*	*0%*	*0 (3)*	*0%*	*0 (3)*	*0%*
*con*	*1 (26)*	*3.8%*	*0 (0)*	*0%*	*1 (26)*	*3.8%*
con	6 (49)	12.2%	2 (18)	11.1%	8 (67)	11.9%
con	6 (76)	7.9%	1 (29)	3.4%	7 (105)	6.7%
con	3 (28)	10.7%	1 (32)	3.1%	4 (60)	6.7%

Total	40 (686)	4.6%	9 (183)	4.9%	49 (869)	5.6%

^*∗*^PE: professional experience; res: resident; con: consultant; numbers in italics: low volumes.

**Table 5:** PTH rates broken down by the N-/R-classification and age groups.

Age	TE_trad_				TE_mic_				Total
Class	N0	N1	R0	R1	N0	N1	R0	R1	
0–10	1 (0.15%)	0	0	1 (0.15%)	0	0	0	0	2 (0.23%)
11–18	0	5 (0.73%)	1 (0.15%)	3 (0.44%)	0	1 (0.12%)	0	0	5 (0.58%)
19–65	3 (0.44%)	16 (2.34%)	1 (0.15%)	7 (1.02%)	1 (0.55%)	5 (2.75%)	0	1 (0.12%)	34 (3.93%)
>65	0	2 (0.29%)	0	0	0	0	0	1 (0.12%)	3 (0.35%)

Total	4 (0.59%)	23 (3.37%)	2 (0.29%)	11 (1.61%)	1 (0.55%)	6 (3.30%)	0	2 (1.10%)	49 (5.66%)
